# Comparison of AI-generated radiology impressions: a multi-stakeholder evaluation

**DOI:** 10.1038/s41746-026-02586-6

**Published:** 2026-04-04

**Authors:** Sharang Phadke, Nivedita Suresh, Zachary Allen, Anjali Balagopal, Stephen Chan, Anish Shah, Megan Winter, Cesar Lam, Trevor Rose, Cyrillo Araujo, Abraham Ahmed, Iman Imanirad, Lincoln Berland, Andrew Del Gaizo

**Affiliations:** 1 Rad AI, San Francisco, CA USA; 2 Moffitt Cancer Center, Tampa, FL USA

**Keywords:** Cancer, Health care, Medical research, Oncology

## Abstract

A retrospective, blinded evaluation of 200 oncologic computed tomography reports compared original radiologist-authored impressions, impressions generated by a custom domain-specific AI model fine-tuned on institutional data, and impressions generated by a general-purpose large language model. Ten clinicians, including original radiologists (*n* = 4), independent radiologists (*n* = 3), and oncologists (*n* = 3), rated impressions for completeness, correctness, conciseness, clarity, clinical utility, and patient harm. Original and independent radiologists assigned lower preference to generic model impressions (Cohen’s h 1.04–1.22 and 0.66–0.69, *p* < 0.001). Original radiologists slightly preferred their own impressions to the custom model (*h* = 0.18, p = 0.0716), while independent radiologists showed no preference (*h* = −0.03, p = 0.78). Oncologists demonstrated no significant preference among impression types (*h* = 0.04–0.12, all *p* > 0.20). Custom model impressions achieved near parity with human impressions; original radiologists rated their own impressions slightly more complete (*r* = 0.22, *p* = 0.0016). Generic model impressions were longer (75.1 ± 20.4 words), slightly more complete (*r* = 0.18–0.39, *p* < 0.001–0.01), but significantly less concise (*r* = 0.85–0.87, *p* < 0.001). Patient harm ratings were uniformly low (likelihood 1.01–1.14; extent 1.05–1.21). Inter-rater reliability ranged from −0.09 to 0.67 (*α* = 0.67 conciseness; *α* = −0.09–0.03 clinical utility/correctness).

## Introduction

The radiology impression represents the most critical component of diagnostic imaging reports, serving as the primary communication mechanism between radiologists, ordering clinicians, and patients. These summaries distill complex imaging findings into actionable clinical insights that guide patient management decisions. However, generating high-quality impressions requires significant cognitive effort and time, contributing to radiologist burnout amid increasing case volumes and workforce shortages^1,2^.

Beyond their immediate clinical role, radiology impressions also represent a high-value source of structured data for secondary use. Standardized, data-rich impressions support imaging biobanks and precision medicine by enabling large-scale aggregation of imaging findings, automated extraction of imaging biomarkers, and population-level analyses, a potential further amplified by AI and large language models that can generate and harmonize structured impressions across heterogeneous reporting styles^3,4^.

Recent advances in artificial intelligence (AI) have demonstrated promising capabilities for automating radiology impression generation. Early foundational work by Zhang et al. demonstrated that neural sequence-to-sequence models could generate impressions that radiologists rated as equivalent to human-authored summaries in 67% of cases^5^. Work by Nakaura et al. showed that the GPT-4 model performed well at drafting radiology reports overall but performed significantly worse than radiologists when drafting the impression and differential diagnosis sections^6^. More recently, commercial AI impression drafting systems have achieved widespread deployment, suggesting technical maturity for real-world implementation and increasing radiologist acceptance.

Despite these technical advances, significant gaps remain in the understanding of AI-based impression generation systems. First, most evaluations of AI tools in radiology reporting focus on assessing entire AI-generated reports rather than specifically evaluating the quality of AI-generated impressions, even though impressions carry elevated clinical importance^7,8^. Second, studies that have focused on evaluating impressions have either been limited to the chest radiograph modality or have applied overly lenient evaluation criteria, such as defining “acceptable” impressions as those requiring major changes but still deemed helpful^7,9^. Finally, existing evaluations largely reflect the radiologist’s initial perspective, overlooking the broader ecosystem of stakeholders who rely on impressions, including ordering clinicians, patients, and downstream radiologists reviewing prior studies for comparison.

This study addresses these gaps by conducting a comprehensive multi-stakeholder evaluation of AI-generated radiology impressions. The quality of impressions from three sources is compared using a real-world dataset of computed tomography reports: original authoring radiologists, a commercially available domain-specific impression generation system, and a general-purpose large language model with high performance on medical benchmarks^10,11^. The evaluation framework incorporates established quality metrics for clinical summarization and patient safety and includes assessment by original authoring radiologists, independent radiologists, and non-radiologist clinicians to capture diverse perspectives across the healthcare delivery process.

## Results

### Impression preferences

**Fig. 1:**
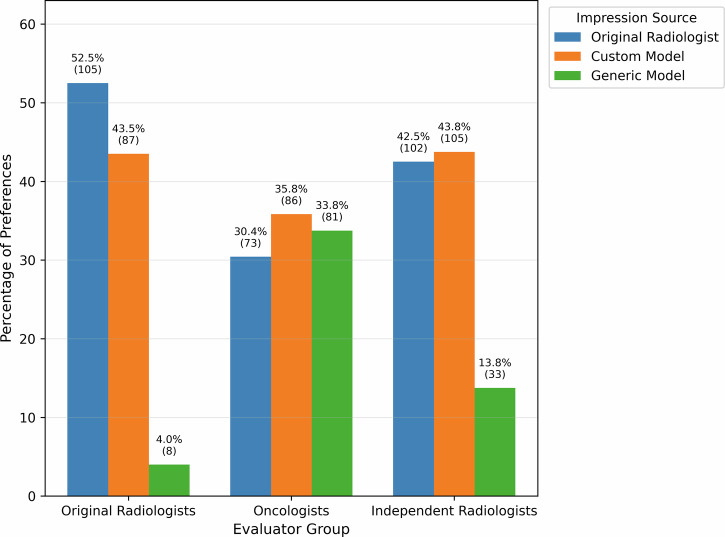
Distribution of impression preferences by evaluator group comparing original radiologist-authored impressions, custom model impressions, and generic large language model impressions.

Preference comparisons differed by evaluator group (Fig. 1**;** Table 1). Differences in Table 1 are computed as (proportion preferring first impression—proportion preferring second impression), with negative values indicating greater preference for the second listed impression. Among original authoring radiologists, original impressions were preferred slightly over domain-specific custom AI impressions with a small effect size that did not reach statistical significance (difference = 0.09; Cohen’s *h* = 0.18; *p* = 0.0716). In contrast, original radiologists demonstrated a strong preference for both original impressions and custom AI impressions over generic model impressions (differences = 0.485 and 0.395; Cohen’s *h* = 1.22 and 1.04; both *p* < 0.001).

**Table 1:** Preference significance analysis *(Two-proportion z-test)*

Evaluator Group	Comparison	Difference (First–Second)	Cohen’s *h*	*p*-value
Original Radiologists	Original vs Custom Model	9.0%	0.18	0.0716
	Original vs Generic Model	48.5%	1.22	< 0.001
	Custom Model vs Generic Model	39.5%	1.04	< 0.001
Oncologists	Original vs Custom Model	−5.4%	-0.12	0.2074
	Original vs Generic Model	−3.3%	-0.07	0.4341
	Custom Model vs Generic Model	2.1%	0.04	0.6318
Independent Radiologists	Original vs Custom Model	−1.3%	-0.03	0.7822
	Original vs Generic Model	28.7%	0.66	< 0.001
	Custom Model vs Generic Model	30.0%	0.69	< 0.001

Differences are calculated as *(proportion preferring the first impression−proportion preferring the second impression)*. Negative values indicate greater preference for the second listed impression (e.g., a negative value for “Original vs Custom Model” indicates greater preference for the Custom Model). Effect sizes are reported using Cohen’s *h*.

Independent radiologists demonstrated no meaningful preference between custom and original AI impressions (difference = 1.3%; Cohen’s *h* = 0.03; *p* = 0.7822). Strong preferences were observed for both original impressions and custom AI impressions over generic model impressions (differences = 28.7% and 30.0%; Cohen’s *h* = 0.66 and 0.69; both *p* < 0.001).

Oncologists showed a slight preference for the custom impression model, though did not reach statistical significance, with effect sizes of 0.12 (*p* = 0.2074), 0.07 (*p* = 0.4341), and 0.04 (*p* = 0.6318) for pairwise comparisons of original vs custom model, original vs generic model, and custom model vs generic model, respectively.

### Quantitative impression characteristics

Quantitative characteristics of impressions are summarized in Table 2. Generic model impressions were substantially longer than both original and custom model AI impressions, with a mean word count of 75.1 ± 20.4 words compared with 41.2 ± 21.4 words for original impressions and 34.2 ± 17.4 words for custom model AI impressions. Generic model impressions also contained more impression items on average (6.3 ± 1.6) than original impressions (2.9 ± 1.2) and custom model AI impressions (3.0 ± 1.2).

**Table 2:** Quantitative Characteristics of Impressions

Impression Type	Word Count (mean ± SD)	Impression Items (mean ± SD)	Generation Time (*s*)
Original	41.2 ± 21.4	2.9 ± 1.2	N/A
Custom model	34.2 ± 17.4	3.0 ± 1.2	1.9 ± 0.8
Generic model	75.1 ± 20.4	6.3 ± 1.6	11.6 ± 3.6

Word count and impression items are reported as mean ± standard deviation (SD). Generation time reflects model inference time in seconds (s). N/A indicates not applicable.

Mean generation time for domain-specific custom model AI impressions was 1.9 ± 0.8 s, compared with 11.6 ± 3.6 s for generic model impressions.

### Impression quality ratings

**Fig. 2:**
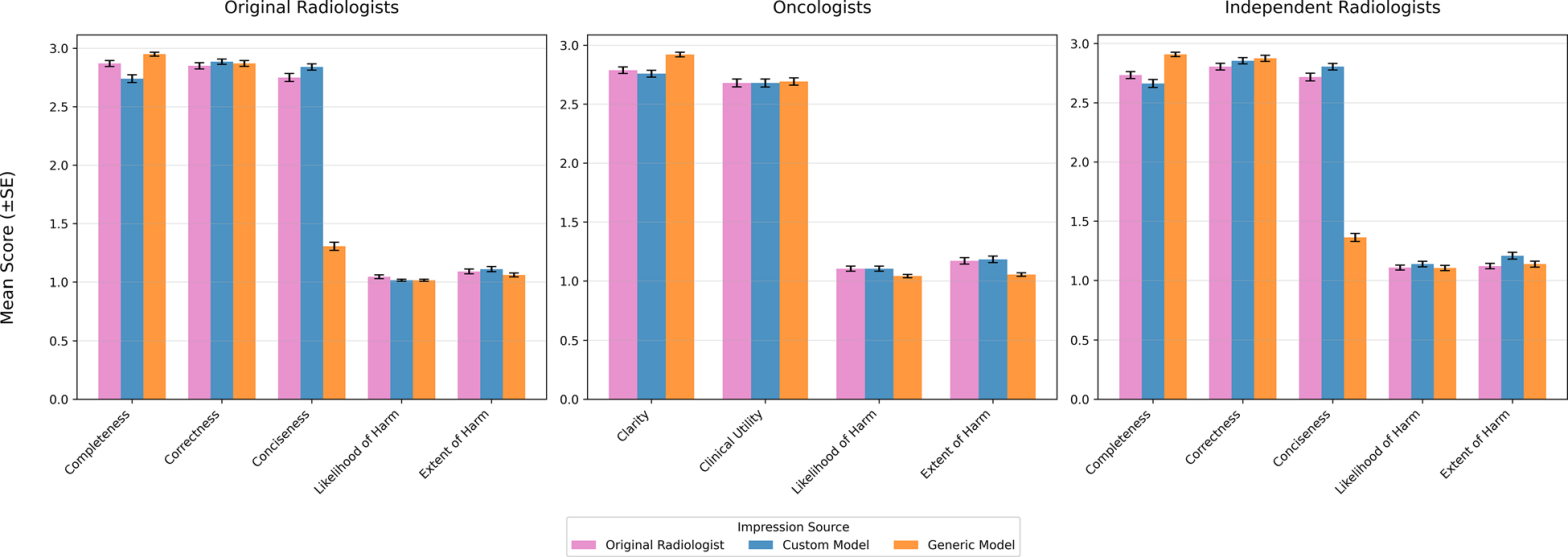
Mean impression quality ratings by evaluator group and impression type across evaluated quality domains.

Mean quality ratings by evaluator group and impression type are shown in Fig. 2 and summarized in Table 3, with pairwise statistical comparisons provided in Table 4. Among original radiologists, original impressions were rated as slightly more complete than custom AI impressions (2.87 ± 0.37 vs 2.74 ± 0.47; *r* = 0.22; *p* = 0.0016). No meaningful differences were observed between original and custom AI impressions for correctness or conciseness. Distributions of qualitative ratings of generic model impressions were found to be meaningfully different from the other two impressions on conciseness and completeness by both groups of radiologists. Generic model impressions were rated as much less concise *r* = 0.85–0.87 (*p* < 0.001), and slightly more complete *r* = 0.18–0.39 (*p* < 0.001–0.01), showing where generic model impressions fall in the trade-off between detail and brevity in the impression writing process.

**Table 3:** Mean Quality Ratings and Patient Harm Scores by Evaluator Group and Impression Type

Evaluator Group	Metric	Original Radiologist	Custom Model	Generic Model
Original Radiologists	Completeness	2.87 (0.37)	2.74 (0.47)	2.95 (0.24)
	Correctness	2.85 (0.39)	2.88 (0.32)	2.87 (0.35)
	Conciseness	2.75 (0.48)	2.84 (0.38)	1.30 (0.49)
	Likelihood of Harm	1.04 (0.23)	1.01 (0.12)	1.01 (0.12)
	Extent of Harm	1.09 (0.29)	1.11 (0.31)	1.06 (0.24)
Oncologists	Clarity	2.79 (0.43)	2.76 (0.45)	2.92 (0.31)
	Clinical Utility	2.68 (0.52)	2.68 (0.53)	2.69 (0.48)
	Likelihood of Harm	1.10 (0.33)	1.10 (0.33)	1.04 (0.22)
	Extent of Harm	1.17 (0.42)	1.18 (0.43)	1.05 (0.23)
Independent Radiologists	Completeness	2.73 (0.46)	2.66 (0.52)	2.91 (0.29)
	Correctness	2.80 (0.44)	2.85 (0.41)	2.88 (0.40)
	Conciseness	2.72 (0.51)	2.80 (0.43)	1.36 (0.52)
	Likelihood of Harm	1.11 (0.32)	1.14 (0.36)	1.10 (0.34)
	Extent of Harm	1.12 (0.35)	1.21 (0.45)	1.14 (0.39)

Values are reported as mean (SD). Completeness, correctness, conciseness, clarity, and clinical utility were rated on 3-point Likert scales (higher scores indicate better quality). Likelihood and extent of harm were rated on 3-point ordinal scales, where 1 indicates none/low harm.

**Table 4:** Pairwise Statistical Comparisons of Quality Ratings *(Wilcoxon signed rank test)*

Evaluator Group	Metric	Original vs Custom Model		Original vs Generic Model		Custom Model vs Generic Model	
		*p*	*r*	*p*	*r*	*p*	*r*
Original Radiologists	Completeness	0.0016	0.22	0.01	0.18	< 0.001	0.39
	Correctness	0.2743	0.08	0.5691	0.04	0.631	0.03
	Conciseness	0.0339	0.15	< 0.001	0.86	< 0.001	0.87
	Patient Harm Likelihood	0.0833	0.12	0.1088	0.11	1	0
	Patient Harm Extent	0.4795	0.05	0.2568	0.08	0.0771	0.12
Oncologists	Clarity	0.8529	0.01	< 0.001	0.26	< 0.001	0.28
	Clinical Utility	0.8242	0.02	0.9329	0.01	0.8918	0.01
	Patient Harm Likelihood	0.9179	0.01	0.0264	0.16	0.0206	0.16
	Patient Harm Extent	0.7265	0.02	< 0.001	0.29	< 0.001	0.28
Independent Radiologists	Completeness	0.103	0.12	< 0.001	0.3	< 0.001	0.37
	Correctness	0.1134	0.11	0.0409	0.14	0.6834	0.03
	Conciseness	0.3479	0.07	< 0.001	0.85	< 0.001	0.85
	Patient Harm Likelihood	0.4659	0.05	0.6311	0.03	0.2855	0.08
	Patient Harm Extent	0.0277	0.16	0.6263	0.03	0.1029	0.12

*P* values and effect sizes (*r*) are reported for each comparison within evaluator groups. Effect size *r* was calculated as Z/√N.

Independent radiologists demonstrated similar patterns, with no meaningful differences between original and custom model AI impressions and significantly lower conciseness ratings for generic model impressions. Oncologists rated clarity and clinical utility similarly across impression types, with generic model impressions rated as slightly clearer but not more clinically useful.

### Patient harm assessment

Across all evaluator groups and impression types, ratings of potential patient harm were low (Table 3). Further, no significant difference was appreciated between human and AI models for either radiologist cohort. Oncologist ratings showed a slight difference among impression sources, with the generic model rated as having a slightly lower extent of harm than the other two impression types, with *r* = 0.28–0.29 (*p* < 0.001) (Table 4).

### Free text analysis

To better understand reasons for qualitative ratings, the 150 out of 680 total ratings that included an optional free text comment were coded for thematic analysis. The most likely reasons motivating preferences were the level of detail in the impression (50%), the clarity, specificity, or prioritization of the impression (24%), other reasons (14%), and the presence of recommendations or clinical inference (12%).

### Inter-rater reliability

Inter-rater reliability analysis on qualitative ratings revealed substantial variability in impression quality assessments. Table 5 shows Krippendorff’s α values and the 95% confidence interval for the 2 annotator groups with overlapping annotator cases, oncologists and independent radiologists. These values indicate low to moderate agreement, suggesting high subjectivity in impression quality assessment even among evaluators with similar clinical backgrounds. Agreement was highest for conciseness among independent radiologists and lowest for correctness and clinical utility.

**Table 5:** Inter-annotator Reliability Analysis using Krippendorff’s alpha

Evaluator Group	Metric	Krippendorff’s alpha (95% CI)
Oncologists	Clarity	0.05 (−0.12, 0.23)
	Clinical Utility	−0.09 (−0.2, 0.01)
	Patient Harm Likelihood	0.07 (−0.07, 0.2)
	Patient Harm Extent	0.12 (−0.04, 0.29)
	Preferred Impression	0.29 (0.05, 0.5)
Independent Radiologists	Completeness	0.3 (0.09, 0.5)
	Correctness	0.03 (−0.1, 0.16)
	Conciseness	0.67 (0.54, 0.76)
	Patient Harm Likelihood	0.05 (−0.09, 0.18)
	Patient Harm Extent	0.07 (−0.07, 0.2)
	Preferred Impression	0.03 (−0.17, 0.23)

Krippendorff’s α values are shown with 95% confidence intervals (CI) for evaluator groups with overlapping cases. Higher *α* indicates greater agreement.

## Discussion

This study represents the first comprehensive multi-stakeholder evaluation of AI-generated radiology impressions, comparing human-authored impressions against two distinct AI approaches: a custom model and a general-purpose large language model. The structured evaluation approach with three groups of blinded evaluators and the use of complex computed tomography reports allows for a thorough understanding of real-world quality considerations and preferences.

Across multiple quality dimensions, domain-specific custom model AI impressions closely matched original radiologist impressions, with only modest differences observed in completeness ratings by original authors and no meaningful differences identified by independent radiologists (Fig. 2**;** Table 3**;** Table 4).

The strong preference among radiologists for original and customized AI impressions over generic model impressions (Fig. 1**;** Table 1) highlights the importance of domain adaptation and stylistic alignment in clinical text generation. The fine-tuning training setup for the custom model likely enabled a combination of personalization to the subjective judgement of the radiologist in question, as well as development of latent radiology knowledge. Meanwhile, generic large language model outputs were substantially longer and more detailed (Table 2), which corresponded to slightly higher completeness ratings but markedly lower conciseness scores (Table 3**;** Table 4). This pattern is consistent with prior evaluations of general-purpose language models in clinical text generation, which frequently demonstrate a tendency toward exhaustive enumeration of findings at the expense of prioritization and signal-to-noise ratio^6,7^. Prior studies by Sun et al. and Nakaura et al. similarly found that generic LLMs produced impressions with lower quality ratings and worse differential diagnoses compared to radiologists, though both used minimal prompting strategies (a single sentence and a short paragraph, respectively) compared to over a dozen best practices for impression generation we outlined in our generic model prompt^7,8^. Our finding that domain-specific fine-tuning closes this quality gap aligns with Van Veen et al., who demonstrated that adapted LLMs can match or exceed medical expert performance on clinical summarization tasks^8^. Domain-specific fine-tuning appears to enable AI systems to internalize local reporting conventions and stylistic norms that balance completeness with efficiency, a characteristic that has been identified as important for clinical acceptance of AI-generated radiology text^5,9^.

Oncologists demonstrated no statistically significant preference among impression types (Fig. 1**;** Table 1) despite clear differences in length and structure (Table 2). Generic model impressions were rated as slightly clearer by oncologists, which may reflect the benefit of additional contextual detail for non-radiologist readers. Similar findings have been reported in prior studies, suggesting that downstream clinicians may value explanatory context and narrative clarity more than brevity alone^7,8^. However, this increased clarity did not translate into higher clinical utility ratings, indicating that verbosity alone does not necessarily enhance decision-making support. This divergence between radiologist and non-radiologist preferences echoes findings by Sun et al., who reported that referring physicians perceived GPT-4-generated impressions as more coherent and less harmful than radiologist-generated impressions, even as radiologists rated the same AI outputs as inferior across all quality metrics^7^. The consistency of this stakeholder divergence across imaging modalities (chest radiographs in their study and oncologic computed tomography in ours) strengthens the argument for adaptive AI systems that accommodate varying clinical perspectives.

Patient harm ratings remained uniformly low across all impression types and evaluator groups (Table 3). No clinically meaningful differences were observed between human-authored and AI-generated impressions among radiologists, consistent with earlier work demonstrating low rates of factual error in constrained radiology summarization tasks^5–7^. Although oncologists rated generic model impressions as having a slightly lower extent of potential harm, the absolute magnitude of this difference was small and unlikely to be clinically meaningful. As in prior studies, harm assessments were based on expert judgment rather than observed patient outcomes and should be interpreted cautiously^12^.

A consistently low inter-rater reliability was observed across multiple quality metrics, including within homogeneous professional groups (Table 5). Agreement was highest for conciseness among independent radiologists and lowest for correctness and clinical utility. This variability has been observed in physician-based evaluations of clinical text summarization and likely reflects legitimate differences in clinical judgment, communication preferences, and interpretive emphasis rather than measurement error^13,14^. These findings challenge evaluation paradigms that assume the existence of a single objective ground truth for impression quality and suggest that consensus-based benchmarks may obscure meaningful dimensions of performance.

Taken together, these results support a shift in how AI-generated radiology impressions are evaluated and deployed. Rather than optimizing models toward a single idealized output, greater emphasis should be placed on adaptability, editability, and alignment with user-specific preferences. AI-generated impressions may be most valuable when positioned as flexible drafting tools that reduce cognitive load and documentation burden while preserving clinician oversight and control. This perspective aligns with emerging informatics frameworks emphasizing human-AI collaboration rather than automation alone^8,11^. More broadly, general-purpose LLMs have demonstrated a range of limitations in radiology, including misinterpretation of domain-specific conventions such as laterality determination and a tendency toward verbose, underprioritized outputs that diverge from clinical reporting norms^15^. A recent editorial in *Radiology* emphasized that lightweight domain adaptation remains more effective than zero-shot prompting of increasingly capable general-purpose models for radiology reporting tasks^16^. While general-purpose models continue to improve in reasoning ability, these advances do not inherently address the stylistic and institutional conventions that shape impression acceptability, which are learned implicitly through exposure to local reporting practices rather than through general medical knowledge.

Future work should extend these findings through prospective, in-workflow evaluations that assess how AI-generated impressions are edited, accepted, or rejected in routine clinical practice. Such studies should examine impacts on reporting efficiency, cognitive workload, downstream clinical decision-making, and patient outcomes. Establishing best practices for safe and effective integration of generative AI into radiology reporting will require continued collaboration between clinicians, informaticians, and AI developers^5,8,11^.

Several limitations should be considered when interpreting these findings. First, this study was conducted at a single academic cancer center and focused exclusively on oncologic computed tomography examinations. Reporting practices, case complexity, and stakeholder expectations at a tertiary referral center may differ from those in community settings or non-oncologic practices, potentially limiting generalizability to other institutions, imaging modalities, or patient populations. Accordingly, these findings should be interpreted as most reflective of academic oncologic computed tomography practice and may not generalize to other settings or modalities.

Second, the evaluation was performed in a retrospective, review-based setting rather than within a live clinical workflow. Evaluators assessed static impressions outside the context of time pressure, competing clinical demands, and downstream communication, which may influence perception of impression quality, conciseness, and utility in routine practice. The study did not measure reporting efficiency, cognitive workload, editing behavior, or real-time acceptance of AI-generated impressions, all of which are critical determinants of clinical value and adoption.

Third, patient harm assessments were based on expert judgment rather than observed clinical outcomes. While this approach is consistent with prior safety evaluations of clinical documentation tools, it may underestimate rare but consequential failure modes that emerge only through longitudinal or real-world use. The low frequency of high-harm ratings in this study should therefore be interpreted as reassuring but not definitive evidence of safety.

Fourth, the generic large language model was evaluated using a standardized zero-shot prompting strategy without domain-specific fine-tuning or institutional adaptation. Although this approach reflects common real-world use of general-purpose models, optimized prompting, context augmentation techniques, or fine-tuning may improve performance and alter comparative results. Conversely, the custom model benefited from exposure to historical reports authored by participating radiologists, which may limit generalizability to settings without access to large volumes of high-quality institutional training data.

Fifth, the small sample size of evaluators in the study, and low to moderate inter-rater reliability across quality metrics may limit the interpretability of aggregate scores. Findings with low p-values in the study may reflect personal preferences of a small number of potentially correlated evaluators, and lack of significant findings in certain comparisons may reflect small sample size and low power that a larger study may have uncovered. That said, our inter-rater reliability findings also reflect the inherently subjective nature of impression evaluation. Differences in clinical judgment, communication preferences, and tolerance for verbosity are intrinsic to radiology reporting and should be considered a feature of the evaluation context rather than solely a methodological limitation. Accordingly, effect sizes are reported alongside p-values to aid interpretation, given the limited number of evaluators.

Finally, this study evaluated impression quality in isolation and did not assess downstream effects on clinical decision-making, diagnostic accuracy, treatment planning, or patient outcomes. Future studies should incorporate prospective, in-workflow evaluations with longitudinal follow-up to better characterize the real-world impact of AI-assisted impression generation.

In summary, this multi-stakeholder evaluation demonstrates that AI-generated radiology impressions can achieve quality and safety comparable to human-authored impressions, particularly when domain-specific fine-tuning is applied. Differences in preference across stakeholder groups and low inter-rater agreement highlight that impression quality is inherently subjective and context dependent rather than defined by a single objective standard. These findings suggest that the clinical value of AI-generated impressions lies not in replacing radiologists, but in supporting adaptable, user-aligned drafting workflows that preserve clinician oversight. Prospective, in-workflow evaluations will be essential to determine how such systems influence reporting efficiency, clinical decision-making, and patient outcomes in real-world practice.

## Methods

### Study design and setting

A retrospective, blinded evaluation study was conducted at a large U.S. academic cancer center (Moffitt Cancer Center, Tampa, FL). The study was approved by the Institutional Review Board of Moffitt Cancer Center under expedited review (protocol number MCC19208). The requirement for informed consent was waived due to the retrospective nature of the study and the use of deidentified radiology reports, which posed minimal risk to participants. The research was conducted in accordance with the ethical standards of the institutional research committee and the principles of the Declaration of Helsinki. All reports were deidentified prior to analysis using a proprietary deidentification pipeline. Clinical trial number: not applicable.

#### Case selection

The study cohort consisted of 200 oncologic computed tomography examinations interpreted by four attending abdominal radiologists. Fifty reports were randomly selected for each radiologist from examinations performed between February 7, 2023, and August 7, 2024. No additional inclusion or exclusion criteria were applied. During the study period, no AI-assisted impression generation tools were in clinical use, and all selected reports were held out from any AI model training or fine-tuning.

### AI Impression Generation

For each original radiology report, two additional impression versions were generated using distinct AI approaches.

The first approach (Custom Model) employed a proprietary, multi-component approach that includes several data transformations and ML models, most notably a radiology-specific impression generation model fine-tuned using 2−5 years of historical reports from each of the participating radiologists (Rad AI, San Francisco, CA).

The second approach (Generic Model) utilized a commercially available general-purpose large language model (GPT-4.1; OpenAI, San Francisco, CA) with a structured, radiology-focused prompt. Zero-shot prompting was employed without in-context examples and incorporated role prompting, stepwise reasoning guidance, and structured output constraints. The final prompt used for the generic model is provided in Supplementary Note 1.

Both AI systems received identical input consisting of the findings section, clinical indication, and imaging protocol information from the original report.

### Evaluation framework

Impressions were evaluated by original authoring radiologists (*n* = 4), independent radiologists (*n* = 3), and oncologists (*n* = 3). Evaluations were conducted at least six months after the data cutoff to minimize recall bias. All evaluators were blinded to the impression source.

The 4 original radiologists were US board certified radiologists with fellowship training in abdominal imaging and between 14 and 24 years of experience. Each was assigned 50 of their own cases, for a total of 200 cases reviewed. Oncologists were US board certified and fellowship trained, with between 4.5 and 13 years of experience. Independent radiologists were US board certified abdominal radiologists with between 3.5 and 30 years of experience. The 3 oncologists and 3 independent radiologists were each assigned a random sample of 80 cases, covering the 200 cases plus 40 double-rated, allowing for sample overlapping to assess rating concordance.

Radiologists rated impressions on completeness, correctness, and conciseness using validated 3-point Likert scales adapted from prior physician review frameworks^13^. All clinician groups rated the likelihood and extent of potential patient harm using previously published adverse event harm rating criteria^12^. Oncologists additionally rated clarity and clinical utility.

In all cases, raters were blinded to the source of each impression. Finally, evaluators selected a preferred impression for each case and could optionally provide free-text comments explaining their preference. Exact questions posed to raters can be found in Supplementary Note 2. While our evaluation framework attempts to structure annotator review along the dimensions above, we explicitly did not have a training and calibration phase for annotators, as we aimed to capture real-world perspectives among different groups of clinicians and avoid instruction bias.

### Statistical analysis

Descriptive statistics were computed for impression length, number of impression items, and AI generation time. Impression items were defined as individual statements within the impression section separated by newline characters. When present, common bullet or numbering characters (e.g., “-”, “•”, or numeric lists) were treated as delimiters for separate items. Each newline-delimited statement was counted as a single impression item. Narrative paragraph-style impressions without newline separation were counted as a single item. Item identification was implemented programmatically using rule-based parsing of newline and bullet delimiters. An example case illustrating the common pattern can be found in Supplementary Note 3.

To compare quality ratings across the three impression types (original radiologist, custom model, generic model) within each reviewer group, pairwise comparisons of quality ratings were performed between impression types using Wilcoxon signed-rank tests. P-value and effect size r (calculated as Z/√N) were calculated for each comparison and interpreted using standard benchmarks (0.1 = small, 0.3 = medium, 0.5 = large). Effect sizes are reported alongside p-values to aid interpretation.

Evaluator preferences were summarized using frequencies and proportions for each impression type, stratified by evaluator group. To test for significant differences in preference between impression types, pairwise two-proportion z-tests were conducted for each pair of impressions within each evaluator group. The effect size using Cohen’s h was reported and interpreted using standard benchmarks (0.2 = small, 0.5 = medium, 0.8 = large).

Optionally added free-text comments were summarized for preferences using inductive thematic analysis. A single coder develops and applies an iteratively established codebook, assigning one or more codes to each comment. Codes are then grouped into higher-level themes for summary analysis.

For the independent radiologist and clinician groups with 40 overlapping cases, inter-rater reliability was calculated per annotator group using Krippendorff’s alpha, a metric that generalizes to multiple annotators, supports ordinal and nominal data, and works for incomplete data^14^. The 95% confidence intervals were computed using bootstrap resampling at the item level with replacement using 2000 samples. We interpret the scores using standard benchmarks: *α* = 1 indicates perfect agreement, *α* > 0.8 strong agreement, 0.67−0.8 moderate agreement, *α* < 0.67 poor agreement, and *α* = 0 indicates chance agreement.

All analyses are performed using Python version 3.8 or later. Statistical significance is defined as *p* < 0.05 (two-tailed) for all tests.

## Supplementary information

Supplementary information

## Data Availability

The datasets generated and/or analyzed during the current study are not publicly available due to institutional data use restrictions, but are available from the corresponding author on reasonable request and subject to applicable approvals.
