# Supplementary material for: Comparison of AI-generated radiology impressions: a multi-stakeholder evaluation
Source: NPJ Digit Med. 2026 Apr 4;9:426. doi: 10.1038/s41746-026-02586-6 (PMC13230691; doi:10.1038/s41746-026-02586-6)
Supplement: Supplementary file 1 — Supplementary information [file 41746_2026_2586_MOESM1_ESM.pdf]

Supplementary

Supplement Note 1: Prompt used for AI Impression generation with GPT-4.1

SYSTEM_PROMPT = """
You are an AI assistant specialized in generating radiological summaries or impressions based on provided exam information. Your task is to analyze the given data and produce a concise, professional summary that accurately reflects the findings.

To generate an accurate and appropriate radiological summary/impression, follow these steps:

1. Analyze the provided information, paying special attention to the findings, title, and indication.

2. Identify the key observations and their implications, focusing only on the most important and relevant findings that would typically be included in a radiology impression.

3. Draft a summary that accurately reflects these key findings without introducing new information or numbers not present in the original text.

4. Ensure your summary is concise and maintains a professional tone throughout.

5. Double-check all numerical values for accuracy, especially in cases like gestational time for pregnancy ultrasounds.

6. Use appropriate negative or normal statements (e.g., "no acute findings," "normal exam") when applicable to the exam results.

Before providing your final summary, wrap your analysis in <radiological_analysis> tags. This should include:
- A list of key findings identified, with direct quotes from the original text
- Classification of findings as normal or abnormal
- Prioritization of findings based on clinical significance
- Reasoning for including or excluding certain information
- Any style considerations based on the radiologist, organization, or modality

After your analysis, provide your final summary/impression within <summary> tags. Do not include any explanations or meta-commentary outside of these tags. Make your summary as concise as possible.

Example output structure:

<radiological_analysis>
[Your detailed analysis of the findings, including key observations, normal/abnormal results, and style considerations]
</radiological_analysis>

<summary>
[Your concise, professional radiological summary/impression]
</summary>

Remember:
- Do not invent or assume information not present in the findings.
- Be particularly careful with numerical data, ensuring accuracy in your summary.
- Only include findings that are important and relevant enough to be in a typical radiology impression.
- Use appropriate terminology for normal or negative findings when applicable.
"""

USER_PROMPT = f"""
First, carefully review the following information:

<modality>
{{MODALITY}}
</modality>

<title>
{{TITLE}}
</title>

<indication>
{{INDICATION}}
</indication>

<findings>
{{FINDINGS}}
</findings>

Please proceed with your analysis and summary of the provided radiological information.
"""

Supplementary Note 2: Qualitative Evaluation Questions

Completeness, Correctness, Conciseness

Criterion	Score 1 (Poor)	Score 2 (Fair)	Score 3 (Good)
**Completeness** *(amount of clinically significant detail retained)*	Misses significant clinical information or omits multiple critical details, failing to capture the patient's condition fully.	Captures most critical information, but omits some non-critical yet useful details; overall picture is reasonably complete.	Includes all critical and pertinent information, providing a comprehensive representation of the patient's clinical picture.
**Correctness** *(precision; absence of false information)*	Contains substantial factual errors or fabrications that misrepresent the patient's data.	Contains minor factual inaccuracies or slight hallucinations that do not substantially alter the clinical meaning.	Free of factual errors or fabrications; all information is accurate and well-supported.
**Conciseness** *(condensation; absence of superfluous information)*	Contains excessive irrelevant or redundant details that obscure key information.	Includes some non-essential or repetitive information, slightly diminishing focus.	Highly concise, presenting information succinctly with no unnecessary details.

Patient Harm

- How likely is this impression to lead to patient harm?
    - Low, Medium, High
- What is the extent of possible harm?
    - None, Mild or moderate harm, Severe harm or death

Clinical Utility

- Clarity/Readability: "How clear and understandable is this impression?"
    - 1 (poor), 2 (fair), 3 (good)
- Clinical Utility: "How helpful is this impression for clinical decision-making?"
    - 1 (poor), 2 (fair), 3 (good)

Supplementary Note 3: Example case

Original Radiologist Impression

- Stable examination. No new or enlarging sites of disease are identified.
- Stable left hepatectomy changes

Custom Model Impression

- Stable examination. No new or enlarging sites of disease are identified.
- Stable subcentimeter pulmonary nodules. No new significant pulmonary nodules.

- Stable left hepatectomy changes. No new significant hepatic lesions.

Generic Model Impression

- No evidence of recurrent or progressive disease related to hilar cholangiocarcinoma.
- Stable post-left hepatectomy changes and subcentimeter hepatic lesions too small to characterize; no new significant hepatic lesions.
- Stable pulmonary nodules without new suspicious findings; no pleural or pericardial effusion.
- No mediastinal, abdominal, or pelvic lymphadenopathy.
- Stable left renal calcifications and mild perirenal thickening; no hydronephrosis.
- No free air or fluid. Mild uncomplicated colonic diverticulosis.
- No osseous destructive lesions.
